# Therapies Targeting Trained Immune Cells in Inflammatory and Autoimmune Diseases

**DOI:** 10.3389/fimmu.2020.631743

**Published:** 2021-01-25

**Authors:** Cristina Municio, Gabriel Criado

**Affiliations:** Grupo de Enfermedades Inflamatorias y Autoinmunes, Instituto de Investigación Hospital 12 de Octubre (i+12), Madrid, Spain

**Keywords:** trained immunity, autoimmune disease, inflammation, therapy, metabolism, epigenetics, COVID-19

## Abstract

The concept of trained immunity has recently emerged as a mechanism contributing to several immune mediated inflammatory conditions. Trained immunity is defined by the immunological memory developed in innate immune cells after a primary non-specific stimulus that, in turn, promotes a heightened inflammatory response upon a secondary challenge. The most characteristic changes associated to this process involve the rewiring of cell metabolism and epigenetic reprogramming. Under physiological conditions, the role of trained immune cells ensures a prompt response. This action is limited by effective resolution of inflammation and tissue repair in order to restore homeostasis. However, unrestrained activation of innate immune cells contributes to the development of chronic inflammation and tissue destruction through the secretion of inflammatory cytokines, proteases and growth factors. Therefore, interventions aimed at reversing the changes induced by trained immunity provide potential therapeutic approaches to treat inflammatory and autoimmune diseases like rheumatoid arthritis (RA). We review cellular approaches that target metabolism and the epigenetic reprogramming of dendritic cells, macrophages, natural killer cells, and other trained cells in the context of autoimmune inflammatory diseases.

## Introduction

Vertebrate immunity is coordinated by a complex interplay of physical and chemical barriers (epithelia and antimicrobial substances), humoral factors and different cell types that reacts to the continuous exposure to diverse internal and external stimuli. Traditionally, the responses to these challenges have been classified as two independent systems, innate and adaptive immunity.

Innate immunity has been classically defined as a rapid and nonspecific response that comprises biochemical and cellular mechanisms that are present before infection and are considered the first line of defense. It is triggered within minutes after infection but does not generate immune memory because their effectors are germline-encoded. Its main components are the complement system, myeloid cells (neutrophils, monocytes, dendritic cells, and macrophages), natural killer (NK) cells or innate lymphoid cells (ILCs), responsible for molecular recognition and antigen presentation, phagocytosis and elimination of pathogens. In contrast, adaptive immunity is carried out by B and T lymphocytes and has been described as slow and specific. It takes days or weeks to generate an adequate humoral and cellular response, mediated by genetic rearrangement and clonal selection. This results in antigen-specific responses that can induce lasting immune memory.

This dichotomy has changed in recent years and both immune arms are currently considered highly intertwined and collaborative. The dogma establishing the innate system as nonspecific and incapable of adaption or develop immunological memory has been replaced by a model where phagocytosis, microorganism removal or lysis of infected cells are considered as one more specific response. This has been due, at least in part, to the discovery of Pathogen Associated Molecular Patterns (PAMPs), essential microbial components, and the endogenous signs of damage (Damage-Associated Molecular Patterns, DAMPs), which are recognized through the constitutive expression of different families of Pattern-Recognition Receptors (PRRs) (e.g., Toll-like receptors, NOD-like receptors, C-type lectin receptors, RIG-I-like receptors) thus allowing the implementation of an adequate response depending on the type of molecules that are recognized (1–4).

## The Concept of Trained Immunity

Mackaness, G.B (5). described in mice that, in addition to generating specific B and T cell memory upon exposure to one pathogen, there is an increase in the innate response that can generate cross-protection against a second pathogen, regardless of its phylogenetic origin. This finding was then attributed solely to CD8 memory lymphocytes. Recently, the issue has sparked renewed interest and multiple works have focused on the activation state of innate immunity against a stimulus and the cross-protection that is generated against a second challenge. This type of “memory” against past inflammatory events is well established in plants and is known as Systemic Acquired Resistance (SAR) (6, 7). In that case, plants that are inoculated with attenuated microorganisms develop lasting protection against a wide spectrum of plant pathogens (8). Therefore, SAR is considered a form of innate memory in plants equivalent to immunization in vertebrates. It is also been described in a variety of invertebrates, ideal animal models to study innate immunity as they do not present adaptive immunity: insects such as *Anopheles, Drosophila*, and the mealworm beetle *T. molitor* (9, 10), nematodes such as *C. elegans* (11); or corals (12). This situation allows us to be prepared for future challenges and supposes an ancestral form of “immune memory”.

Similarly, “a heightened response to a secondary infection that can be exerted both toward the same microorganism and a different one (cross-protection)” has been termed “innate immune memory” or “trained innate immunity” in vertebrates (13).

Exposure of innate immune cells to a stimulus through PRRs, promotes a series of long-term modifications that involve rewiring of cell metabolism and epigenetic reprogramming. Since several metabolites function as signalling molecules or cofactors for the enzymes responsible of epigenetic changes, these two processes are closely related (14). Depending on the type and concentration of PAMPs, this immunological imprint can lead to two opposite outcomes: trained innate immunity or innate immune tolerance. In the case of trained immunity, the “training” generates a greater response to a second challenge, while innate immune tolerance is aimed at attenuating or reducing this response (14). Thus, strategies aimed at potentiating the latter can be very useful in regulating physiological processes to avoid harmful reactions to allergens, the microbiota or autoimmune inflammation. However, there must be a balance between the pro- and anti-inflammatory responses to avoid situations of chronic inflammation or immunoparalysis and increased sensitivity to secondary infections.

Most cells use aerobic respiration as their main source of ATP under homeostatic conditions. In the case of the cells of the immune system, there are important metabolic differences depending on the cell type or its activation state (14). Whereas neutrophils have a high basal glycolytic metabolism, other cell types, such as pro-inflammatory macrophages or T cells, need a rapid increase in their glucose consumption and ATP generation when stimulated. This demands cause a metabolic shift from oxidative phosphorylation to aerobic glycolysis, allowing cells to quickly obtain energy and metabolites. Among the processes necessary for the induction of trained immunity, the following can be considered: the increase of the metabolic capacity of the cells, through the Akt/mTOR/HIF1α/pathway; the accumulation of certain metabolic intermediates of the tricarboxylic acid cycle (TCA) with immunomodulatory functions such as fumarate or succinate (15). Some of these metabolites control histone methylation and acetylation, and others are cofactors for histone and DNA methyltransferases and demethylases, as well as histone acetyltransferases and deacetylases (16).

Recent studies suggest that other metabolic pathways also play an important role in cell reprogramming, such as the fatty acid synthesis pathway, which produces cellular stress and activates innate immunity responses. For example, cellular accumulation of unsaturated fatty acids (oleic acid, linoleic acid) induces a pro-inflammatory phenotype in macrophages due to uncoupling of mitochondrial respiration and production of inflammasome components such as IL1-α (17). Likewise, accumulation of mevalonate derived from the pathway of cholesterol synthesis is related to epigenetic changes that promote trained immunity (18). A role for oxLDL in the induction of trained immunity through the activation of the NLPR3 in monocytes has also been shown by studies analysing the effect of western diet in systemic inflammatory diseases (19). This triggers an inflammatory response and the reprogramming of granulocyte monocyte precursor cells (GMPs) (18–20).

MiRNAs provide an additional layer of regulation in the maintenance of innate immune memory. Due to their stability and long half-life, once induced by a stimulus they are capable of maintaining gene expression programs that enhance the resistance of cells to subsequent insults (21). Some miRNAs, such as miR-146, decrease the activation of NF-κB by blocking TRAF6 and IRAK1 thus limiting the immune response (22). In contrast, miR-155, when activated *via* inflammatory cytokines or TLR ligands, rapidly increases its expression and acts as an activator of inflammation through the down regulation of phosphatases of various signalling pathways (23).

One of the main objections to substantiate the existence of innate immune memory has come from the short half-live of myeloid cells, between 5–7 days, that make difficult to explain how trained immunity is maintained for months or years. However, it has been recently documented that metabolic changes and epigenetic modifications also induce long-term phenotypic and functional reprogramming in the hematopoietic precursors of myeloid cells (HSPC) (24–26). This allows epigenetic reprogramming carried out in innate memory to be transferred through the hematopoietic pathway and its cellular progenitors. Likewise, acquisition of immune functions by fibroblasts and other resident cells can play a role in sustaining organ-specific trained immunity (27, 28).

## Targeting Trained Immunity in Inflammatory and Autoimmune Diseases

The origin and development of autoimmune diseases is mainly attributed to an excessive and sustained response to autoantigens mediated by B and T cells. On the other hand, they are also characterized by excessive inflammation and there is strong evidence that innate immunity reprogramming is one contributing factor.

**Figure 1:**
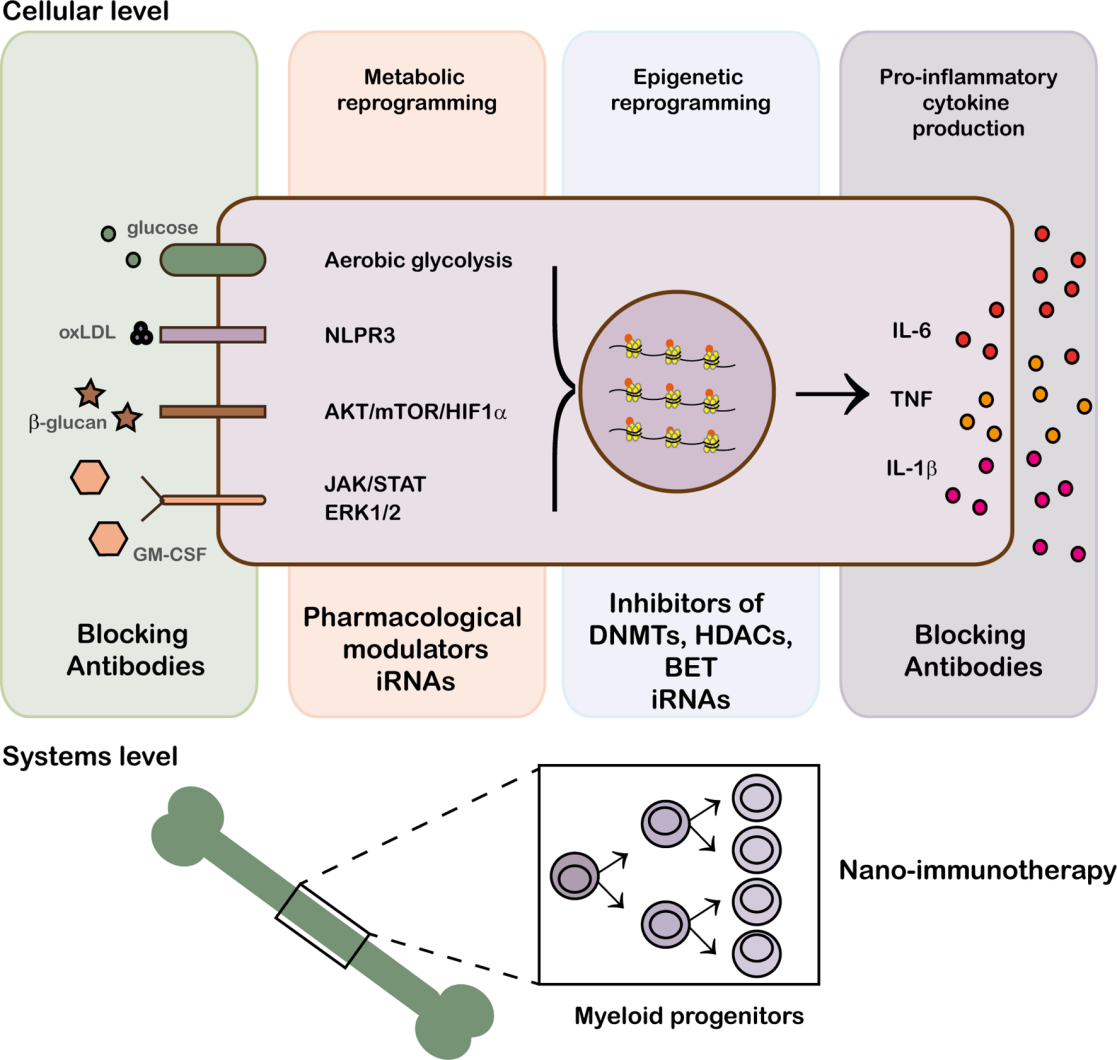
Modulation of trained immunity at epigenetic, cellular and system levels. Trained immunity can be modified at different levels: blockade of receptor recognition with biological therapies, drug modulation of metabolic pathways and epigenetic remodeling.

Under physiological conditions, the action of the innate immune response is curtailed by an effective resolution of inflammation and the induction of tissue repair but when the system is dysregulated and the cellular response gets uncontrolled chronic inflammation and tissue destruction, mediated by inflammatory mediators (cytokines, proteases, growth factors), ensue (29). In this context, it is relevant that, in addition to microbial products, trained immunity can be induced by endogenous stimuli and environmental agents, like tobacco smoke, microbiota, and diet, that contribute to the development of inflammatory and autoimmune diseases (19, 30, 31). Consequently, it has been suggested that strategies aimed at controlling the hallmarks of trained immunity, i.e., altered metabolism and epigenetic reprogramming, can provide potential treatments for the chronic inflammation associated to autoimmunity (**Figure 1**) (29).

### Manipulation of Metabolic Pathways

The shift from oxidative phosphorylation to aerobic glycolysis is a critical component of reprogramming trained immunity and there are several pharmacological modulators that target glucose metabolism. 2-DG (2-deoxy-D-glucose) and 3-BP (3-bromopyruvate), that block glycolysis by inhibiting pathway-limiting enzymes, have shown protection in animal models of arthritis and Systemic lupus erythematosus (SLE) (32, 33). Their effects are mainly attributed to the action on T cells and stromal cells, but they can also regulate the numbers and activity of myeloid cells in model of inflammatory arthritis (34, 35). In line with this observation, oxamate, an alternative inhibitor of glycolysis, reduced the proinflammatory polarization of human macrophages *in vitro* (36). Lack of tissue specificity and concerns about their toxicity limit the application of these glycolytic inhibitors.

Conversely, the fact that mTOR inhibitors, such as metformin and rapamycin, are already used in the clinic for the treatment of transplant rejection and glycemic control make them more attractive candidates to target trained immunity. Metformin synergizes with 2-DG in the treatment of lupus mice (33) and has shown protective effects in some models of arthritis and Sjöegren syndrome (37–39), but these studies are focussed on acquired immunity. In humans, clinical trials have shown that metformin ameliorates SLE activity, at least in part by reducing neutrophil activation and plasmacytoid dendritic cell function (40, 41).

As in the case of glycolytic inhibitors, the lack of cell specificity makes difficult to evaluate an intrinsic effect on trained immunity. To circumvent this limitation, the application of HDL-based nanobiologics has been used successfully to target mTOR inhibitors to macrophages in the context of transplantation (42).

The lipid metabolism that is involved in trained immunity can be regulated at different levels: treatment with cytochalasin D to block the internalization of CD36, receptor for oxLDL (which induces trained immunity) (20); treatment with methyl-β-cyclodextrin to prevent the formation of cholesterol crystals; inhibition of the enzyme HMG-CoA reductase with fluvastatin to block cholesterol synthesis (18). The inflammasome pathway and the production of IL-1β can be targeted by suppressing NLRP3 activation with Z-VAD-FMK (19), the inhibitor MCC950o, and the ketone metabolite β-hydroxybutyrate (43). To prevent epigenetic changes orchestrated by mevalonate, the enzyme 3-hydroxy-3-methyl-glutaryl-coenzyme A (HMG-CoA) reductase can be inhibited with statins. Several studies have proposed the use of eicosanoid precursors such as omega-3 polyunsaturated fatty acids (PUFAs) as candidates for the treatment of type 1 diabetes, SLE or RA. These molecules have anti-inflammatory properties (44, 45). Also modulation of lipoxins, resolvins and protectins with aspirin is used in the treatment of SLE (46). *In vitro* and *in vivo* studies show how the administration of products of arachidonic acid metabolism (EET, epoxyeicosatrienoic acids) can serve as a therapeutic strategy in those diseases where osteoclastogenesis is deregulated, such as rheumatoid arthritis (RA) (47).

### Epigenetic Therapy

Reverting the epigenetic modifications that occurred during the immunity training to normal values ​​using different inhibitors can potentially be used for the treatment of inflammatory and autoimmune diseases (48). Inhibitors of DNMTs (DNA methyltransferases) such as azacytidine and decitabine have been used in the field of oncology for 50 years as cytostatics, and recently, as inhibitors of DNMTs, but little is known about their effect outside this field. A wide variety of proteins capable of lysine methylation, a mark associated with transcriptionally silenced chromatin, are potential pharmacological targets of small inhibitory molecules (49). There is also a large number of compounds used as HDACs (histone deacetylase) inhibitors: from molecules pan-HDAC inhibitors, such as trichostatin A (TSA) or vorinostat (SAHA), to other family-specific, such as valproic acid (VPA), givinostat (ITF2357), or etinostat. Regardless of the mechanism, HDAC inhibitors modify the immune response by increasing and decreasing gene expression (48). The effect of multiple HDAC inhibitors in reducing systemic inflammation and pro-inflammatory cytokines has been investigated in various animal models such as arthritis, diabetes, sepsis, asthma. Most of the studies in humans have focused on the context of the RA. The production of proinflammatory cytokines derived from the macrophages of the inflamed synovium can be inhibited by TSA, vorinostat and “sodium phenylbutyrate” (50). Both TSA and MI192 inhibit IL-6 production in LPS-stimulated PBMCs (51). Furthermore, TSA and givinostat (ITF2357) interfere with the stability of IL-6 mRNA, reducing its production in synovial fibroblasts and macrophages (52) and inducing RA synovial fibroblasts to a TRAIL-induced apoptosis (53). Other molecules such as romidepsin (FK228) or MPT0G009 inhibit the proliferation of synovial fibroblasts (54, 55) and FK228 inhibits angiogenesis in synovial tissue (56). Preliminary studies in human monocytes and macrophages show that the small inhibitory molecules of BET bormodomain-containig proteins have great therapeutic potential in the treatment of immune-mediated diseases (48).

### Biological Therapies

Biologics currently used in the clinic for the treatment of autoimmune diseases can also have an impact on trained immunity. For example, Lin and colleagues have described that TNF inhibitors etanercept and adalimumab suppress the expression of CC-chemokine ligand 2 (CCL2) in monocytes by regulating histone acetylation and trimethylation, changes that correlate with RA activity (57).

IL-1β associated with increased inflammasome activity may also serve as a target to actively suppress trained immunity. The observation that monocytes from patients with autoimmune and autoinflammatory diseases showed increased released of IL-1β than healthy individuals (58, 59) promoted IL-1β neutralization as a potential therapy for some chronic diseases. Although the IL-1Ra anakinra is a marginal treatment in RA (60), both anakinra and the IL-1 blocking antibody canakinumab are effective in suppressing symptoms and keeping the disease under control in systemic autoinflammatory syndromes (61, 62). In the case of the “cryopyrin-associated periodic syndrome” (CASP) where there is a mutation in an amino acid that codes for the cryopyrin protein (currently known as NLRP3), early diagnosis along with treatment with an IL-1 blocker is essential to prevent future disabilities or complications (59, 63). It is also approved for use in the treatment of Hyper-IgD syndrome (HIDS). This disease presents a defect in the enzyme mevalonate kinase that favors the AKT/mTOR pathway and the consequent change to glycolytic metabolism. This, together with attacks of sterile inflammation, is a clear example of uncontrolled trained immunity (18, 64, 65) and IL-1 blockers can reduce the frequency and severity of flares (66, 67).

Granulocyte-macrophage colony-stimulating factor (GM-CSF) is a major cytokine in the development of trained immunity (26). Several studies in patients with inflammatory diseases show elevated levels of GM-CSF in blood and synovial fluid, as well as expression of GM-CSFR in inflamed synovial tissues (68, 69). GM-CSF has a main effect in promoting inflammation and therapies aimed at inhibiting its activity are expected to impact trained immunity. Currently, clinical trials addressing the effect of monoclonal antibodies against GM-CSF (namilumab, MOR10) and against GM-CSFR (Mavrilimumab) are underway in patients with RA (NCT02393378; NCT01023256) or psoriatic arthritis (NCT02129777).

## Trained Immunity and COVID-19

It has been proposed that trained immunity can be beneficial against SARS-CoV2 infection (70). In this context, trained immunity induced by BCG vaccination protects from several viral infections (71, 72). However, attempts to find an association between BCG vaccination status and COVID-19 severity have yielded inconclusive results (73, 74).

Conversely, the presence of previous inflammatory diseases and the excessive inflammatory response triggered by SARS-CoV2 infection are poor prognostic factors in COVID-19 progression (75) and, given the role of trained immunity in the development of both processes, its responses can contribute to greater COVID-19 severity (70).

## Concluding Remarks

The reprogramming of the innate immune memory can provide an excellent therapeutic target in autoimmune inflammatory diseases, allowing to restore the balance between hyperinflammation and immunodepression and to achieve therapeutic benefits. However, there are limitations to the *in vivo* use of molecules that target myeloid cells and their progenitors. The compounds used to regulate trained immunity show toxicity, low bioavailability and some adverse effects related to immunity. More suitable approaches are needed to reduce side effects and increase specific targeting.

## Author Contributions

GC and CM conceived the review, searched databases, and drafted the manuscript. All authors contributed to the article and approved the submitted version.

## Funding

This work was supported by the Ministerio de Ciencia e Innovación to CM (Programa Juan de la Cierva IJCI-2016-27666).

## Conflict of Interest

The authors declare that the research was conducted in the absence of any commercial or financial relationships that could be construed as a potential conflict of interest.
